# Immortalization of common marmoset monkey fibroblasts by *piggyBac* transposition of *hTERT*

**DOI:** 10.1371/journal.pone.0204580

**Published:** 2018-09-27

**Authors:** Stoyan Petkov, Tobias Kahland, Orr Shomroni, Thomas Lingner, Gabriela Salinas, Sigrid Fuchs, Katharina Debowski, Rüdiger Behr

**Affiliations:** 1 Platform Degenerative Diseases, German Primate Center- Leibniz Institute for Primate Research, Göttingen, Germany; 2 DZHK (German Center for Cardiovascular Research), Partner Site Göttingen, Göttingen, Germany; 3 Microarray and Deep-Sequencing Core Facility, University Medical Center Göttingen (UMG), Göttingen, Germany; 4 Department of Human Genetics, University Medical Center Hamburg-Eppendorf, Hamburg, Germany; University of Newcastle, UNITED KINGDOM

## Abstract

Following a certain type-specific number of mitotic divisions, terminally differentiated cells undergo proliferative senescence, thwarting efforts to expand different cell populations *in vitro* for the needs of scientific research or medical therapies. The primary cause of this phenomenon is the progressive shortening of the telomeres and the subsequent activation of cell cycle control pathways leading to a block of cell proliferation. Restoration of telomere length by transgenic expression of telomerase reverse transcriptase (TERT) usually results in bypassing of the replicative senescence and ultimately in cell immortalization. To date, there have not been any reports regarding immortalization of cells from common marmoset (*Callithrix jacchus*), an important non-human primate model for various human diseases, with the use of exogenous human *TERT* (*hTERT*). In this study, marmoset fibroblasts were successfully immortalized with transposon-integrated transgenic *hTERT* and expanded *in vitro* for over 500 population doublings. Calculation of population doubling levels (PDL) showed that the derived *hTERT*-transgenic lines had significantly higher proliferation potential than the wild-type fibroblasts, which reached only a maximum of 46 doublings. However, the immortalized cells exhibited differences in the morphology compared with the control fibroblasts and transcriptome analysis also revealed changes in the gene expression patterns. Finally, the karyotypes of all *hTERT*-transgenic cell lines showed various aberrations such as presence of extra Chromosome 17, isochromosome 21q, or tetraploidy. By single-cell expansion of the least affected monoclonal immortalized line, one sub-clonal line with normal karyotype was established, suggesting the possibility to derive immortal marmoset cells with normal karyotypes. The results of this study are an important step towards the development and optimization of methods for the production of immortalized cells from common marmoset monkeys.

## Introduction

The expansion of terminally differentiated somatic cells *in vitro* for research or medical therapy applications is limited by their finite proliferative lifespans. Following a certain cell type-dependent number of mitotic divisions, most cells enter a phase of replicative senescence [1]. The main reason for this restriction is the progressive shortening of telomere ends [2] leading to genomic DNA damage and activation of p53/p21-mediated cell cycle control pathways [3–6]. In some cases, the cells may overcome this replication block due to defective or virus-suppressed p53 and Rb function [7, 8]; however, continued proliferation results in further shortening of telomeres, extensive chromosome damage, and genomic crisis leading to widespread apoptosis [5, 9]. Restoration of the telomere length by telomerase, a ribonucleoprotein complex comprised of internal template RNA (TR) and a specific telomerase reverse transcriptase (TERT), leads to bypassing the crisis and, ultimately, to immortalization [10]. Normally, somatic cells express TR ubiquitously [11], but TERT is either absent or present at very low levels in senescent somatic cells [12] and is therefore the prime determinant of telomerase activity. Various studies have demonstrated that overexpressing exogenous human *TERT* (*hTERT)* in the form of a transgene is sufficient to immortalize various cell types in the human [13–20] as well as in different animal species such as sheep [21], dog [22], pig [23], and rhesus macaque [24]. While some groups have reported relatively normal phenotypes for *hTERT*-immortalized cells [16, 18–20], others have shown that such cells may exhibit cancer-associated changes and neoplastic transformation [25–28]. Nevertheless, immortalization with *hTERT* may have advantages over using tumor-inducing viruses or their components, such as Eppstein-Bar virus [29] or SV40 Large T antigen [30], which have been shown to cause malignancies and genomic aberrations.

There have been only a few reports regarding the immortalization of somatic cells from common marmoset (*Callithrix jacchus*) *in vitro*, despite the importance of this New World non-human primate as a relevant animal model for various research fields that include (but are not limited to) neuroscience, toxicology, reproduction, autoimmunity, and genome editing [31–33]. Using different subgroups of Herpesvirus Saimiri (HVS), two groups described the derivation of immortalized cells from marmoset peripheral blood lymphocytes (PBL) [34, 35]. It was shown that HVS subgroups A and C efficiently immortalized PBLs, while subgroup B showed reduced efficiency and the cells required medium supplementation with IL-2 for continuing proliferation [34]. However, the proliferation time of the cells in this study was limited to only two months. In the other report, HVS was able to immortalize a restricted subset of the marmoset PBL population expressing mainly T8 and NK-associated antigen NKH1 [35]. Another study reported the immortalization of ovarian marmoset cells using the large T antigen [36]. The karyotypes of the derived immortal lines were not examined in any of these reports and it is presently unclear whether these methods of immortalization are adequate to preserve the genomic integrity of the original cell populations over long-term culture.

To our knowledge, no reports have been published regarding immortalization of common marmoset somatic cells *in vitro* by overexpression of exogenous *hTERT*. The aim of this study was to generate immortalized common marmoset cells by stable non-viral, reversible integration and expression of *hTERT* into newborn fibroblasts using the *piggyBac* transposon system. Three of the derived immortalized fibroblast lines were characterized according to morphology, proliferation dynamics, and gene expression.

## Materials and methods

### Animals and procedures

The animals and the procedures used to obtain experimental fibroblasts were described previously [37], as the cells used for *hTERT* immortalization were the same we used earlier for reprogramming to iPS cells [37]. Skin biopsies were acquired from two neonatal common marmosets originating from triplet births that could not be nourished adequately by their mothers and were euthanized to avoid death due to malnutrition. All animal work was performed by experienced veterinarians and trained staff in agreement with the requirements of the German animal protection law (Deutsches Tierschutzgesetz, §6). The German Primate Center is authorized and registered by the local and regional veterinary governmental authorities (reference number 122910.3311900, PK Landkreis Goettingen). Animal procedures to obtain different developmental stages of marmoset monkeys were approved by an external ethics committee (Niedersächsisches Landesamt für Verbraucherschutz und Lebensmittelsicherheit, AZ 42502–04–12/0708). The internal ethics committee of the German Primate Center also approved the care and euthanasia of neonatal marmosets originating from triplet births under the license number E5-17. The selection criteria for euthanasia were the decrease of the health status (body tension, inability to cling to the parent’s coat, low body weight–only animals with continuously decreasing body weight relative to their litter mates were used), and eventually, their expected low survival chances, based on assessments by veterinarians and experienced caretakers. All marmosets were under the care of the primate husbandry facility of the German Primate Center (https://www.dpz.eu/en/unit/animal-husbandry/primates-at-the-dpz/common-marmoset.html). The marmosets were first narcotized with Pentobarbital (0.05 ml Narcoren administered intramuscularly) and then euthanized by intracardial injection of 0.5 ml Pentobarbital prior to taking samples. These animals were not subjected to any previous studies and no samples were taken before the euthanasia took place. The skin biopsies were collected by a veterinarian who delivered them to the tissue culture lab where they were immediately processed by some of the authors (TK and KD).

### Isolation of primary fibroblasts

The skin pieces were washed in 1 x PBS (Life Technologies) and remaining hairs were removed using razor blades. The fragments were finely minced with scalpel blades and digested in Collagenase IV (Gibco) solution dissolved in PBS at final concentration of 5 mg/ml at 37°C for at least 1 h. The cells were centrifuged at 300 x g for 5 min at room temperature, the supernatants were discarded and the cell pellets were disaggregated in M10 medium (DMEM supplemented with GlutaMAX, Penicillin-Streptomycin, and 10% FBS), seeded in 5 cm culture dishes and cultured at 37°C with 5% CO_2_. All of the M10 components were purchased from Gibco. The culture medium was changed twice a week. At approximately 90% density the cells were split following disaggregation with TrypLE Express (Gibco).

### Generation of the pTT-PB-hTERT-puro plasmid and nucleofection of primary fibroblasts

The pBABE-neo-hTERT plasmid was a gift from Dr. Bob Weinberg (Addgene plasmid # 1774) and was the source of the *hTERT* cDNA. The pTT-PB-SOKMLN-puro as well as pcA3-PBase-Tomato (for transient expression of *piggyBac* transposase) plasmid were described previously [37]. The pTT-PB-SOKMLN-puro was used as the backbone for the *piggyBac* transposon after excision of the SOKMLN cassette with EcoRI and SalI. All restriction enzymes and DNA ligase were purchased from New England Biolabs (NEB). The pBABE-neo-hTERT was digested with *Eco*RI and *Sal*I and the DNA fragment containing the *hTERT* cDNA was ligated into the pTT-PB-puro (without SOKMLN). The resulting plasmid pTT-PB-hTERT-puro was transformed into NEB10-beta competent E.coli cells. Afterwards, plasmid DNA was isolated using Maxiprep kit (Qiagen) according to the manufacturer’s instructions.

Common marmoset primary fibroblasts were nucleofected with pTT-PB-hTERT-puro and pcA3-PBase-Tomato using AmaxaTM 4D NucleofectorTM (Lonza) with the P2 Kit for primary mammalian fibroblasts (Lonza). For each nucleofection, 1 x 10^6^ cells were suspended in 100μL buffer P2 together with 9 μg of pTT-PB-hTERT-puro and 6μg pcA3-PBase-Tomato plasmid and nucleofected using program CA-137. The cells with integration of pTT-PB-hTERT-puro were selected with Puromycin (1 μg/ml) for the first 10 passages.

### Derivation of clonal and sub-clonal cell lines

Cells were detached from culture dishes and disaggregated to single cells with TrypLE as described above. Single cells were picked with a fine glass pipette and seeded at one cell per well into 24 well plates. For establishment of sub-clonal lines from one of the derived monoclonal lines (K#1), the cells were disaggregated to single-cell suspension, counted, and seeded at estimated density of 0.5 cells/well in 96-well plates. Ten wells with proliferating cells were randomly chosen for further expansion and karyotype analysis.

### Population-doubling level (PDL) calculation

For determining PDL, the fibroblasts were passaged in 10 cm culture dishes (Thermo) with TrypLE at density of 2,5x10^5^ cells /dish. When reaching confluency, cells were harvested and counted before being seeded again at 2,5x10^5^ cells /dish for the next passage. Outgoing from consecutive counts, the PDL was calculated as follows: PDL(n/n-1) = log (Nf/N0)/log 2, where n = passage number, Nf = final number of cells, N0 = number of cells seeded at passage [38].

### Immunofluorescence

To confirm transgenic hTERT protein expression, we performed immunofluorescence (IF) staining on the *hTERT-* immortalized cell lines as well as non-transfected fibroblasts and ES cells as controls. Prior to the beginning of the procedure, the subjected cells were detached with TrypLE and seeded in 48-well plates. After reaching an appropriate density, the cells were fixed in 4% PFA (in PBS) for 10–30 min and permeabilized with 0.1% TritonX-100 for 10–15 min at room temperature. Following double wash with PBS, the cells were incubated with primary antibody for hTERT (Abcam, Cat. # ab32020; diluted 1:200 in PBS + 5% BSA) overnight at 4°C. Afterwards, the cells were washed twice with PBS and incubated with the secondary antibody, AlexaFluor594 donkey anti-rabbit (Life Technologies, #A21207; diluted 1:200 in PBS + 5% BSA) for 20 min at room temperature in the dark and subsequently stained with 5% DAPI in PBS for 1–2 min at room temperature. Finally, the cells were washed twice with PBS and mounted with Citifluor mountant medium (CITIFLUOR). Microscopy images were taken with a Zeiss Observer Z1 (Zeiss).

### Reverse transcription (RT) and polymerase chain reaction (PCR)

Total RNA was isolated from *hTERT*-immortalized or control fibroblasts either with the NucleoSpin RNA Plus Kit (Macherei-Nagel), or the RNeasy Mini Kit (Qiagen). Both kits were used according to manufacturer’s instructions. The isolated total RNA was reverse transcribed into cDNA using the Omniscript RT Kit (Qiagen) according to the manufacturer’s instructions. PCR reactions were carried out with KOD Hot Start Polymerase (Merck) using a T3000 Thermo Cycler (Biometra). The primer sequences used to amplify the hTERT were: G0957(fwd): CTGGACGATATCCACAGGGC and G0958(rev): AAGTTCACCACGCAGCCATA. As an internal PCR control, beta-actin was amplified using the following primer pair: G0336 (fwd): GACGACATGGAGAAGATCTGG and G0337 (rev): GGAAAGAAGGCTGGAAGAGTG.

### Karyotyping

Proliferating fibroblast cultures were transported to the Cytogenetic Laboratory in the Department of Human Genetics at the University Medical Center Hamburg-Eppendorf (Germany), where the cells were processed according to the standard procedures described previously [37]. Chromosome classification based on G-banding was performed according to the nomenclature presented by Neusser et al. [39]. ]. The following cultures / lines were analysed: one control wild type fibroblast culture, one polyclonal immortalized line, and two monoclonal immortalized lines.

Three of the sub-clonal lines derived later from monoclonal line K#1 were processed at the German Primate Center with the purpose of determining the chromosome numbers of the cells. The cells were treated with 0.02 mg/ml Demecolcine (Sigma) for 3 hours, harvested with TrypLE and incubated in hypoosmotic 0.56% KCl solution for 20 minutes. Following the incubation, the cells were fixed in methanol:acetic acid (3:1) and metaphase spreads were produced by dripping the cell suspension on glass slides positioned at a slight angle over a steaming water bath. The glass slides were dried at 50°C overnight and stained in Giemsa solution (Sigma) for 10 minutes. The metaphase spreads were photographed with Axiophot microscope (Zeiss) equipped with CRI Nuance multispectral imaging camera and 15–30 images per sub-clonal line were used for chromosome counting.

### Transcriptome analysis

Transcriptome analysis was performed by the Transkriptomanalyselabor, Microarray and Deep-Sequencing Facility, University Medical Center Göttingen, as described previously [37]. Six separate cultures from each group (K#1 A-F, K#4 A-F, hTERT-poly A-F, and WT A-F) were used for the transcriptome analysis; three samples from each group consisting of two mixed cultures of the same cell line (e.g. AB, CD, EF) were submitted for RNA-sequencing. Gene ontology analysis of genes upregulated in line K#1 relative to the control was performed using Panther Classification System Version 12.0 (pantherdb.org) based on the gene names of differentially expressed transcripts. The RNA-seq data generated in this study has been deposited in NCBI’s Gene Expression Omnibus database and are accessible through GEO Series accession number GSE114965.

## Results

### Exogenous hTERT prolongs cell proliferation potential in common marmoset fibroblasts

**Fig 1:**
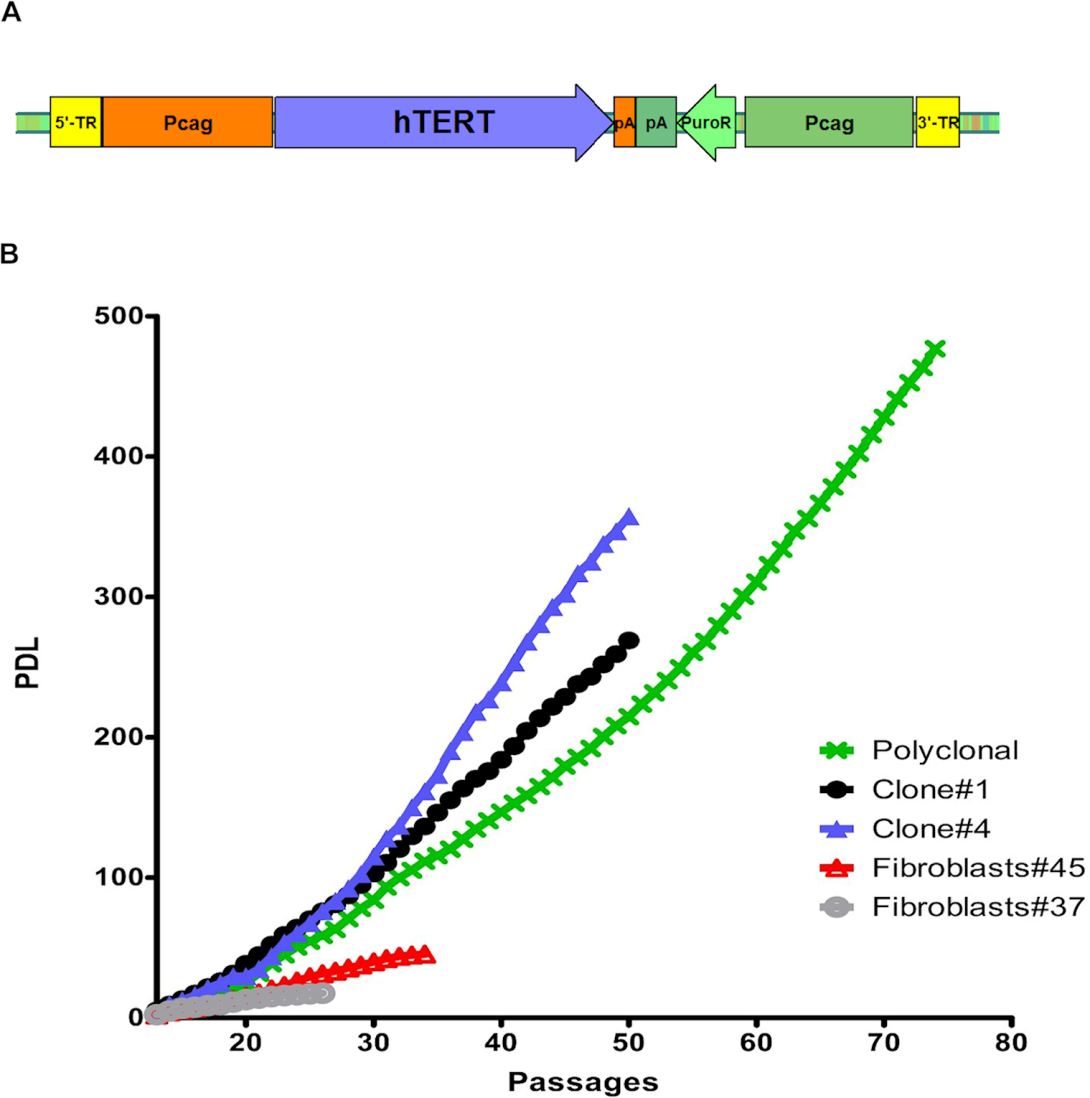
pTT-PB-hTERT-puro plasmid map and population doubling levels (PDL) of immortalized marmoset fibroblasts. (A) The *piggyBac* construct used for immortalization. Human *TERT* is under control of the CAG promoter (Pcag). pA: poly A signal; 5ˈ-TR: 5ˈ-terminal repeat; 3ˈ-TR: 3ˈ-terminal repeat; PuroR: puromycin resistance gene. The latter one is also under the control of a separate CAG promoter. (B) Cell population doubling levels (PDL). PDL was determined according to equation: PDL(n/n-1) = log (Nf/N0)/log 2; n = passage number, Nf = final number of cells, N0 = number of cells seeded at passage.

To immortalize common marmoset fibroblasts, we used CAG promoter-driven *hTERT* (Fig 1A), which was inserted into the marmoset genome by *piggyBac* transposition. Puromycin selection resulted in the establishment of two polyclonal (*hTERT*-poly) and five monoclonal fibroblast cell lines (K#1—K#5). Two of the monoclonal cell lines (K#1 and K#4) were chosen for long-term maintenance in parallel with one polyclonal cell line and the non-transfected primary fibroblasts, which served as negative controls.

To examine whether the *hTERT-*transgenic cells had acquired an extended ability to replicate, we calculated the population-doubling level (PDL) to assess their proliferation potential. The same analysis was performed for the control cells. The *hTERT-*transgenic cells clearly showed higher proliferation potential compared with wild type primary fibroblasts (Fig 1B), as they underwent several hundred population doublings until the experiments were terminated. In contrast, the primary fibroblast controls underwent a significantly lower number of population doublings, reaching only a PDL of 46 and 18, respectively (Fig 1B). These experiments were performed over a period of more than nine months.

### Morphology of *hTERT*-immortalized marmoset fibroblasts

**Fig 2:**
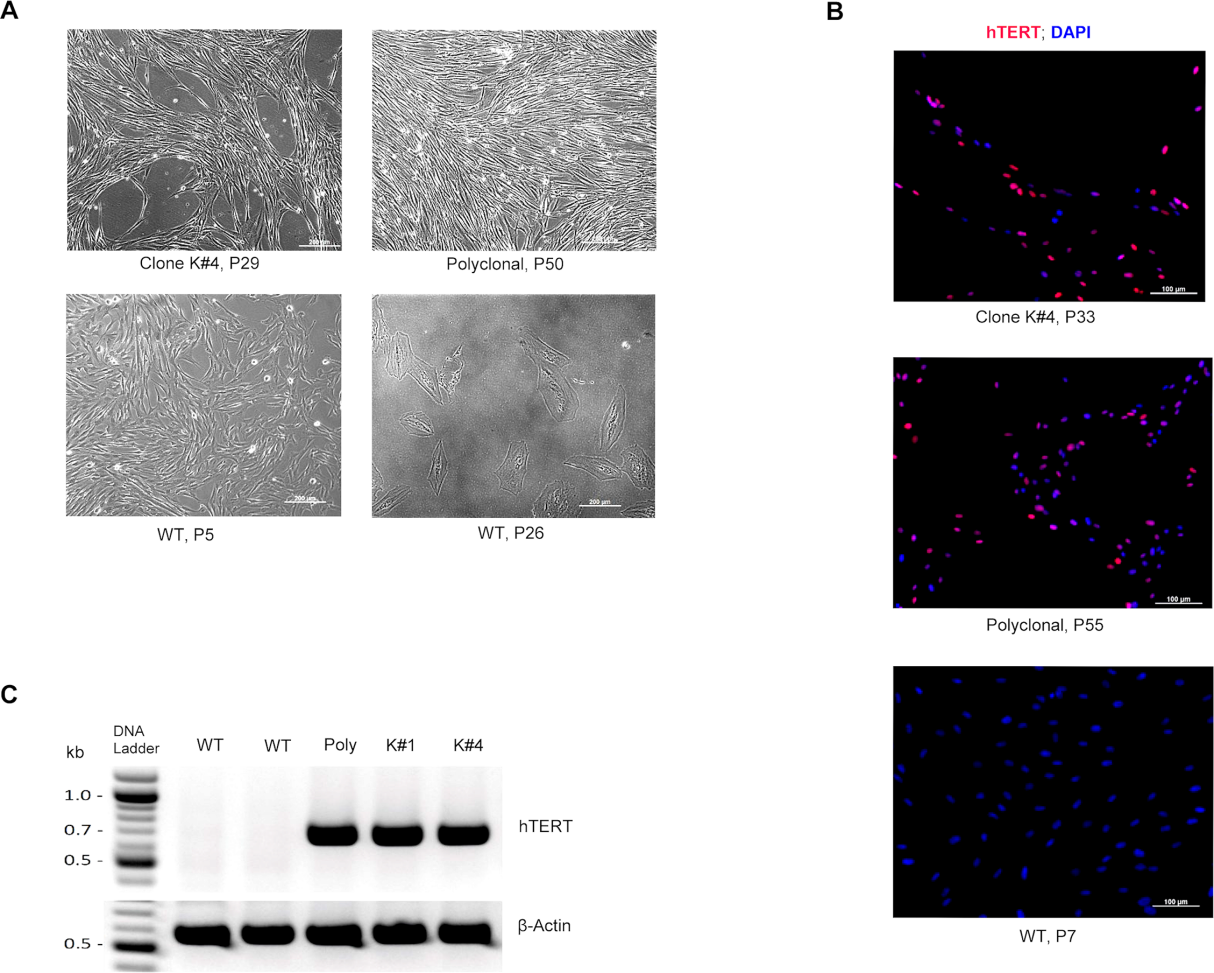
Transgenic hTERT expression. (A) Phase-contrast images of the *hTERT-* immortalized cell lines (upper row) and wild type (WT) controls (lower row). Monoclonal K#4 and the polyclonal cell line show an elongated, “needle like” morphology when compared to WT fibroblasts. Fibroblasts at P26 show a flat and enlarged morphology typical for cells in senescence. Scale bars = 200 μm. (B) hTERT immunofluorescence staining of immortalized cell lines. Immortalized cell lines show expected hTERT protein expression (red). No expression was detected in control fibroblasts. Scale bars = 100 μm. (C) RT-PCR for expression of hTERT (upper row) and β-actin (lower row) in one polyclonal (poly), two monoclonal lines (K#1 and K#4), and two control WT fibroblast lines.

Phase contrast microscopy of the cells revealed a morphological change of the immortalized cells in comparison with the control fibroblasts. All of the established *hTERT*-transgenic lines exhibited an elongated, “needle like” shape (Fig 2A, upper row), while maintaining their proliferation activity even at high numbers of passages. On the other hand, the control fibroblasts showed typical features of cells in senescence (as described by Blagosklonny [40]), exhibiting an enlargement of the cytoplasm (cell hypertrophy), and change from the fibroblast typical narrow, drawn-out morphology to a wider, rounder phenotype at P26 (Fig 2A, lower row).

### Analysis of transgenic *hTERT* expression

All three analyzed immortalized cell lines (polyclonal, K#1, and K#4) reacted positively with the anti-hTERT antibody (Fig 2B), similarly to the control ES cells (not shown). In contrast, the non-transgenic fibroblasts were negative for hTERT-associated immunofluorescence (Fig 2B). From the DAPI staining, it was observed that the *hTERT-* immortalized cell lines have smaller nuclei than primary fibroblasts (Fig 2B). Furthermore, RT-PCR confirmed *hTERT-*mRNA expression in the immortalized cell lines but not in the wild-type (WT) control fibroblasts (Fig 2C).

### Karyotype analysis

**Fig 3:**
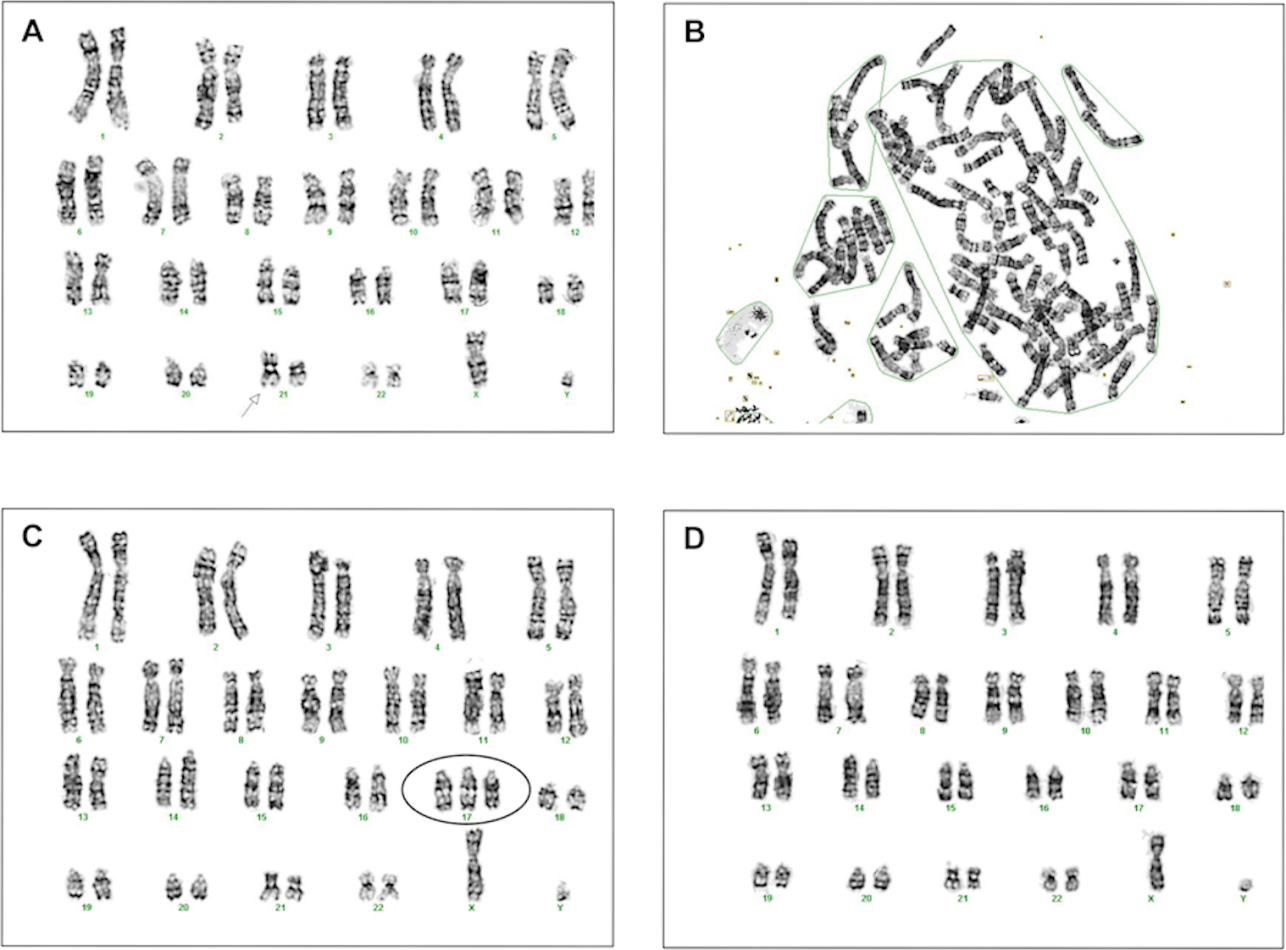
Karyotyping of *hTERT*- immortalized marmoset fibroblasts. (A) Karyotype of polyclonal line hTERT-poly with isochromosome 21q (arrow). (B) Tetraploid karyotype of monoclonal immortalized line K#4. (C) Karyotype of monoclonal line K#1 showing an extra Chromosome 17 (circled) as observed in 6/15 recorded images. (D) Normal diploid karyotype of immortalized monoclonal line K#1 as observed in 9/15 recorded images.

Due to the lack of common nomenclature, it proved difficult to characterize any subtle structural changes in marmoset fibroblast chromosomes based on G-banding. In addition, there are differences in the banding patterns of marmoset chromosomes reported by different authors [39, 41]. In this study, the karyotyping was performed based on the proposed nomenclature by Neusser et al. [39], similarly to another publication [42]. In comparison with the control fibroblast line at low passage with a normal male karyotype 46, XY, all three of the analyzed immortalized lines showed some aberrations. In all of the examined chromosomal spreads (n = 15), the polyclonal line at passage 82 had an abnormal Chromosome 21 with loss of the p-arm and a doubled q-arm, i.e. an isochromosome 21q (Fig 3A). One of the monoclonal lines (K#4), analyzed at passage 56, exhibited tetraploidy in 86/100 examined karyotypes (Fig 3B). In the other monoclonal line (K#1), at the same passage number, 6/15 karyotypes contained an extra Chromosome 17 (Fig 3C) and 9/15 were normal. We established 10 sub-clonal lines from K#1 and performed chromosome counts on 3 sub-clones at passage 98 until we found one sub-clone (K#1.1) to have normal chromosome numbers in all 30 examined metaphase spreads (S1 Fig).

### Transcriptome analysis

**Fig 4:**
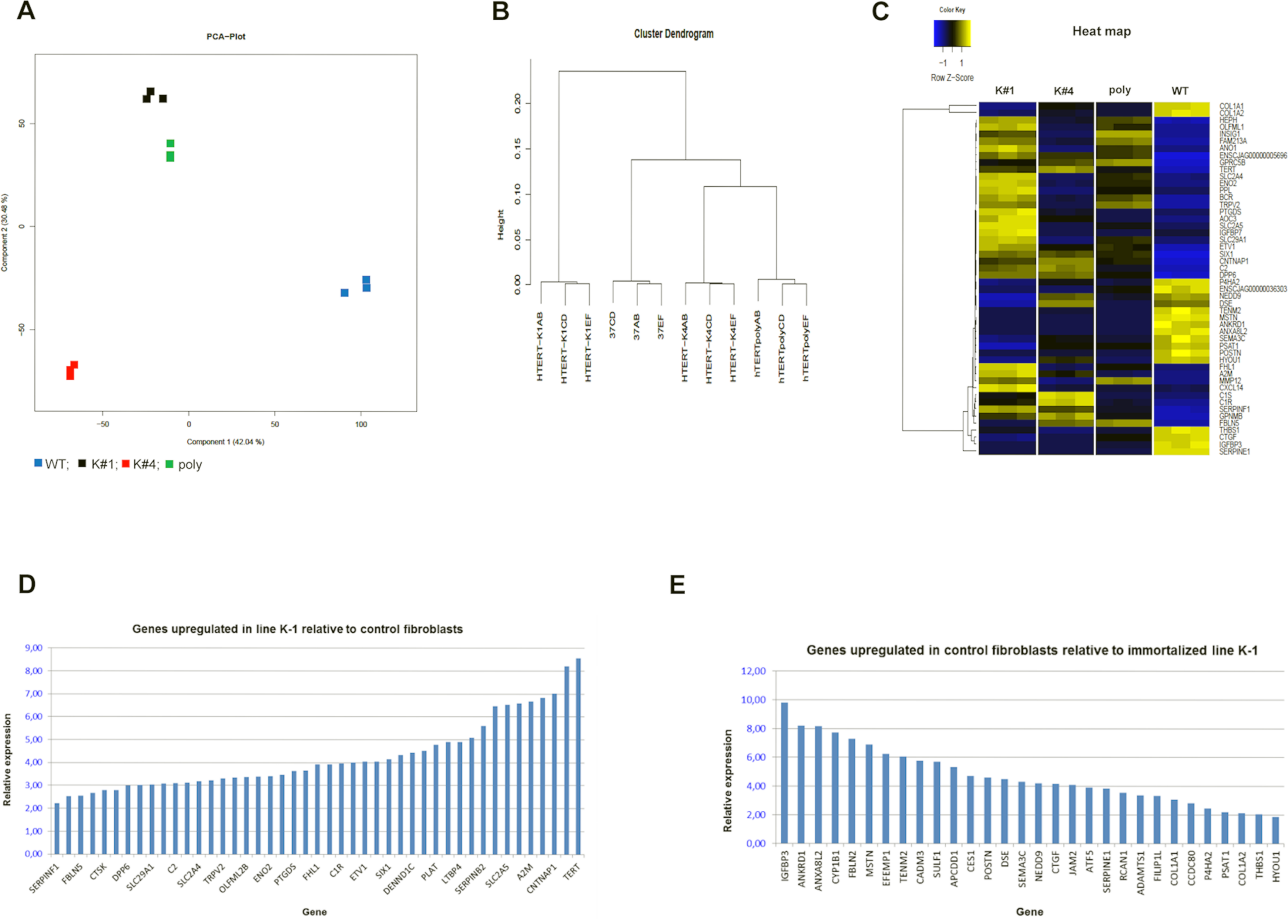
Transcriptome analysis of immortalized marmoset fibroblasts. (A) PCA-plot of a polyclonal (poly), two monocolonal (K#1 and K#4), and one wild-type (WT) control fibroblasts lines. (B) Cluster dendrogram showing that line K#1 clusters farther from the control relative to the other two immortalized lines. (C) Heat map showing the top 50 most significant differences in the gene expression between the WT and K#1. (D) Genes upregulated in immortalized monoclonal line K#1 relative to WT (all p-values = 0.00). (E) Genes expressed higher in WT cells relative to line K#1 (all p-values = 0.00).

The data obtained from the RNA-seq analysis indicate a change in the gene expression patterns in the immortalized cells relative to the control fibroblasts. The larger (42% variance) component 1 of the PCA plot shows separate clustering of the immortalized cells relative to the control (Fig 4A). On the cluster dendrogram (Fig 4B), monoclonal line K#1 clustered farther from the non-immortalized control compared with the other two lines (poly- and monoclonal K#4). The heat map (Fig 4C) shows that line K#1 has altered the expression of multiple genes relative to the control WT cells, such as upregulation of *SERPINF1*, *INSIG1*, *FBLN5*, *CABLES1*, *CTSK*, *ITGB5*, and other endogenous genes, in addition to the exogenous hTERT (Fig 4D). On the other hand, a number of genes were expressed higher in the WT control cells relative to K#1 (in other words: were down-regulated during immortalization), such as *IGFB3*, *ANKRD1*, *ANXA8L2*, *CYP1B1*, *FBLN2*, *MSTN*, *EFEMP1*, *TENM2*, etc. (Fig 4E). Gene ontology analysis (Panther) showed that the genes upregulated in line K#1 are predominantly related to cellular communication and primary metabolic processes (biological process), and catalytic activity, binding, and receptor activity (molecular function) (S2 Fig).

## Discussion

The transposon-mediated transgenic *hTERT* expression in marmoset fibroblasts resulted in significant enhancement of the proliferation capacity and circumvented the Hayflick limit, as evidenced by PDL increase and longer proliferation lifespan of the immortalized cells. Compared to the control cells, which reached maximum of 45 PDL, the immortalized polyclonal line exceeded 500 PDL over the entire period of maintenance in culture without showing any signs of senescence. The sustained expression of *hTERT* in the transfected cell lines was confirmed by immunofluorescence as well as by RT-PCR. However, the immortalized fibroblast lines showed some marked differences compared with the control wild-type fibroblasts, such as reduced nuclear size and changes in cell morphology from typical mesenchymal to elongated, rather needle-like appearance (also at low densities). Transcriptome analysis suggested that these morphological changes were accompanied by a shift in the gene expression patterns as evidenced by the differential clustering of the immortalized cells separate from the non-immortalized control. Gene ontology analysis showed upregulation of genes related to various cellular processes, such as metabolism and cellular communication. While some genes were identified that could contribute to the changed cellular shape like the collagen encoding genes *COL1A1* and *COL1A2*, no gene was found by transcriptome analysis that could directly be linked to the apparently reduced nuclear diameter.

Karyotype analysis revealed that the genomic integrity of the immortalized cells was also compromised in all three of the analyzed lines, with aberrations ranging from structural abnormality of one chromosome to tetraploidy. These results agree with those reported by other groups [26–28]. By performing extensive karyotype analysis of human cells immortalized by overexpression of telomerase, Duesberg and McCormack [26] found that all cell lines had flexible karyotypes similarly to telomerase-deficient clones immortalized with SV40 virus. Based on these results, the authors hypothesized that immortality may be related to the presence of clonal and flexible karyotypes (which could give proliferative advantage to the cells) rather than the maintenance of telomeric lengths. This opinion was later contradicted by Mondello and Chiodi [43], who pointed out that all immortalized cells expressed telomerase and did not show fusion of telomere ends, while the telomerase-deficient primary fibroblasts eventually became senescent, stressing that telomerase expression and immortalization preceded the occurrence of the flexible karyotype. In one of our least severe cases (monoclonal line K#1, which contained an extra Chromosome 17 in part of the cells), we were able to establish a sub-clonal line (K#1.1) with normal chromosomal numbers for 15 passages, suggesting that it might be possible to derive immortalized cells with normal karyotypes. However, it remains to be determined for how much longer this cell line will be able to maintain its integrity and whether further sub-cloning might be necessary to ensure preservation of the normal karyotype characteristics. If cell cultures *in vitro* should represent aspects of normal cellular function and physiology *in vivo*, a normal karyotype is considered essential since aneuploidy is reflected by the transcriptome in human [44] and marmoset cells [45]. Furthermore, postnatal human life is prevented by most trisomies; only trisomies 13, 18 and 21 can be compatible with postnatal survival, although the affected persons exhibit complex syndromes associated with multiple defects, while all other trisomies usually cause early abortions. These embryological and clinical findings emphasize the importance of the use of normal cells in biomedical studies investigating normal physiological conditions.

In conclusion, we successfully established immortalized fibroblast cells from the common marmoset by *piggyBac* transposition of *hTERT*. The derived immortal lines showed morphological and gene expression changes together with flexible karyotypes, suggesting that the method used does not guarantee complete preservation of all original cell characteristics. Nevertheless, as no other method has yet proven qualitatively superior in this respect, this study is a significant step towards the creation and optimization of procedures for generating immortalized marmoset cells.

## Supporting information

S1 Fig Karyotype of sub-clonal immortalized line K#1.1. Total of 30 recorded images were used to determine the karyotype number of the sub-clonal line K#1.1 as 46, XY.
(TIFF)

S2 Fig Panther gene ontology analysis. Genes upregulated in monoclonal immortalized line K#1 are predominantly related to cellular communication and primary metabolic processes (biological process), and catalytic activity, binding and receptor activity (molecular function).
(TIF)
