# Supplementary figures and images for: Immortalization of common marmoset monkey fibroblasts by piggyBac transposition of hTERT
Source: PLoS One. 2018 Sep 27;13(9):e0204580. doi: 10.1371/journal.pone.0204580 (PMC6160115; doi:10.1371/journal.pone.0204580)

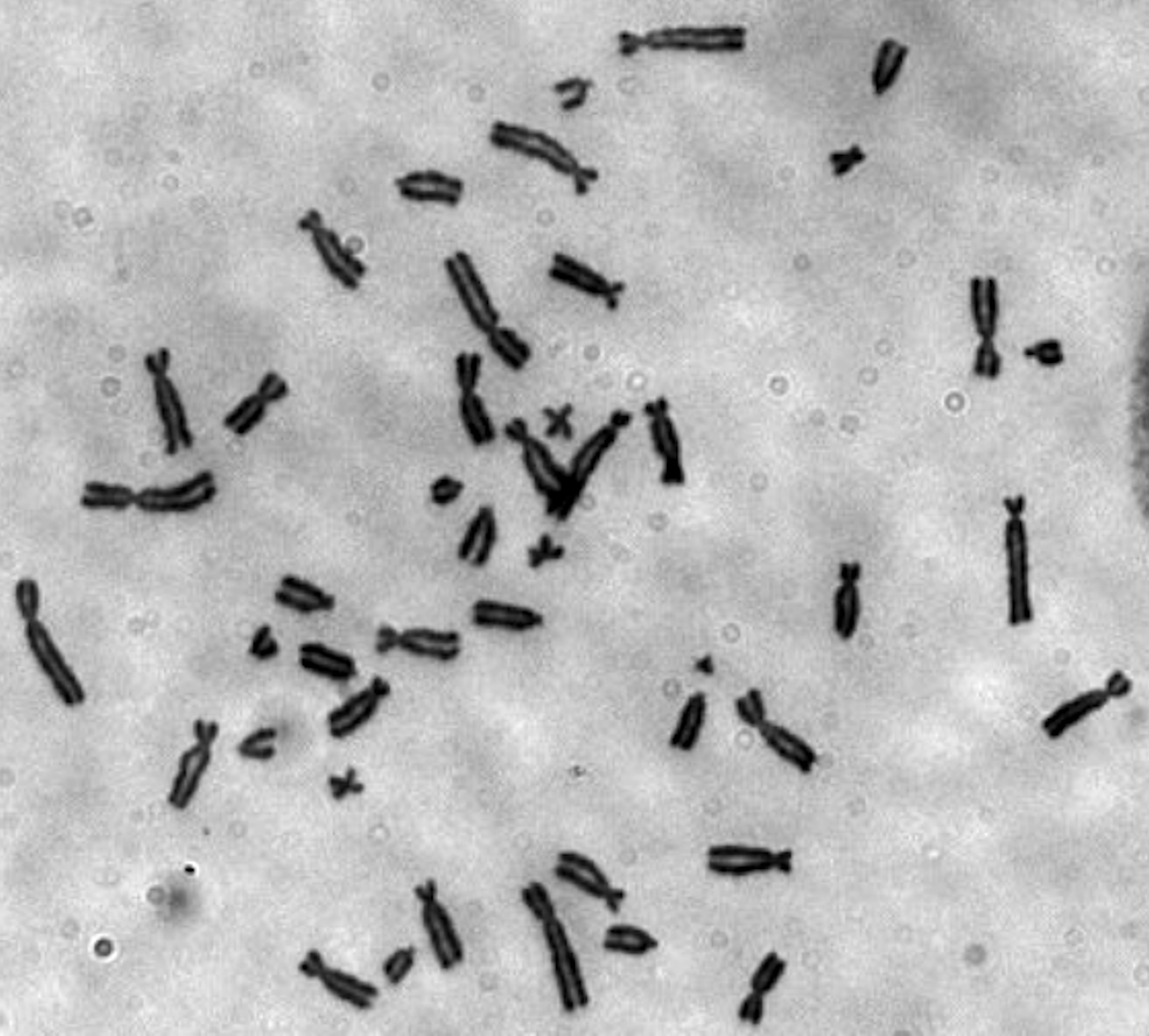

Supplement: S1 Fig — Total of 30 recorded images were used to determine the karyotype number of the sub-clonal line K#1.1 as 46, XY. (TIFF) [file pone.0204580.s001.tiff]

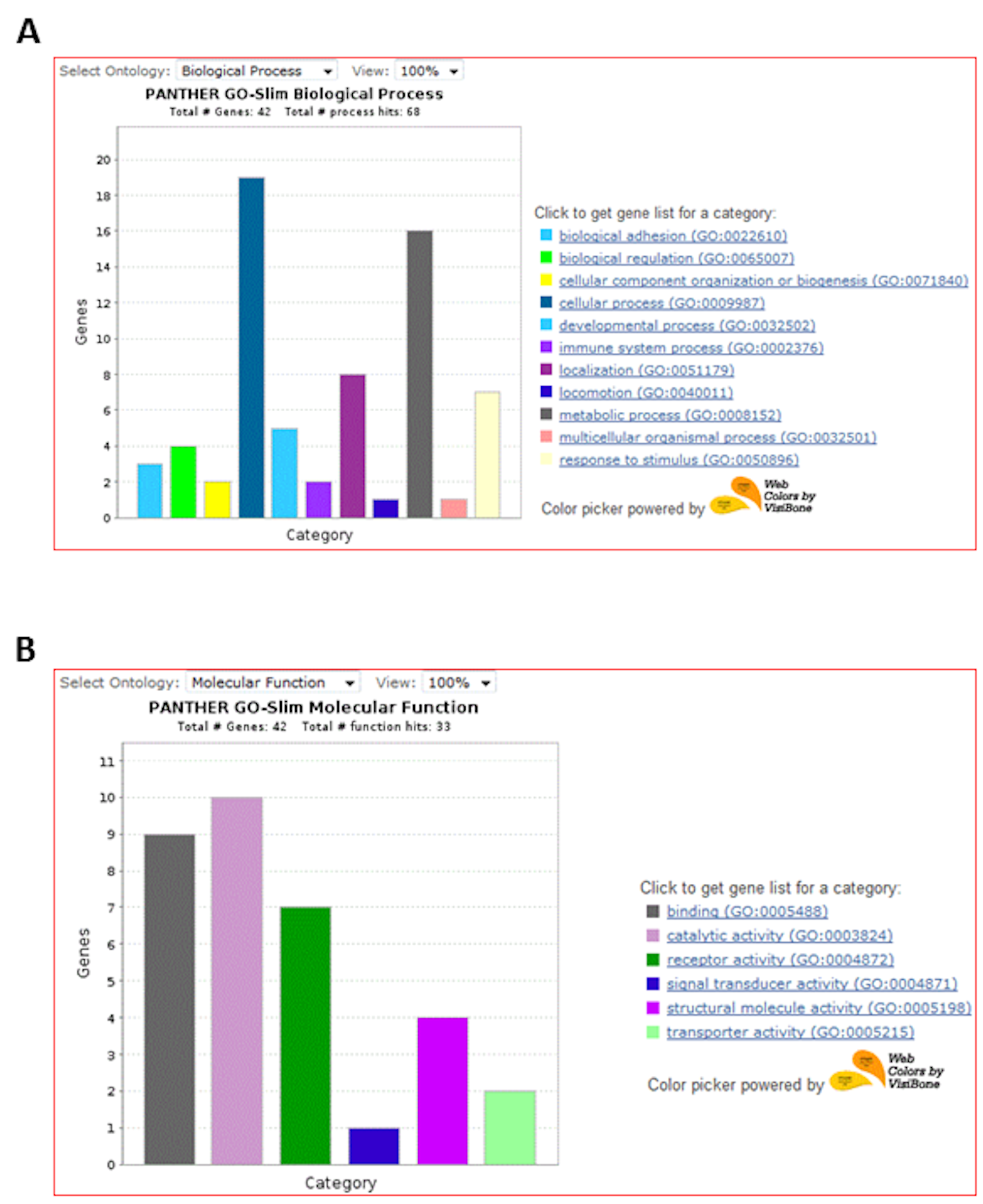

Supplement: S2 Fig — Genes upregulated in monoclonal immortalized line K#1 are predominantly related to cellular communication and primary metabolic processes (biological process), and catalytic activity, binding and receptor activity (molecular function). (TIF) [file pone.0204580.s002.tif]
